# Mori Ramulus (Chin.Ph.)—the Dried Twigs of *Morus alba* L./Part 1: Discovery of Two Novel Coumarin Glycosides from the Anti-Hyperuricemic Ethanol Extract

**DOI:** 10.3390/molecules24030629

**Published:** 2019-02-11

**Authors:** Jianbiao Yao, Houhong He, Jin Xue, Jianfang Wang, Huihui Jin, Jian Wu, Jiangning Hu, Ruwei Wang, Kenny Kuchta

**Affiliations:** 1 Zhejiang Provincial Key Laboratory of Traditional Chinese Medicine Pharmaceutical Technology, Hangzhou 310018, China; yaojb@conbagroup.com (J.Y.); hehh@conbagroup.com (H.H.); xuejin@conbagroup.com (J.X.); wangjf@conbagroup.com (J.W.); jinhh@conbagroup.com (H.J.); wujian@conbagroup.com (J.W.); hujn@conbagroup.com (J.H.) wangrw@conbagroup.com (R.W.); 2 Zhejiang Institute of TCM and Natural Medicine, Hangzhou 310018, China; 3 Clinic for Gastroenterology and Gastrointestinal Oncology, University Medical Center Göttingen, Robert-Koch-Str. 40, 37075 Göttingen, Germany

**Keywords:** *Morus alba* L., coumarin glycosides, structural characterization, electrospray ionization, tandem mass spectrometry

## Abstract

In Traditional Chinese Medicine (TCM), Mori ramulus (Chin.Ph.)—the dried twigs of *Morus alba* L.—is extensively used as an antirheumatic agent and also finds additional use in asthma therapy. As a pathological high xanthine oxidase (XO, EC 1.1.3.22) activity is strongly correlated to hyperuricemy and gout, standard anti-hyperuremic therapy typically involves XO inhibitors like allopurinol, which often cause adverse effects by inhibiting other enzymes involved in purine metabolism. Mori ramulus may therefore be a promissing source for the development of new antirheumatic therapeutics with less side effects. Coumarins, one of the dominant groups of bioactive constituents of *M. alba*, have been demonstrated to possess anti-inflammatory, antiplatelet aggregation, antitumor, and acetylcholinesterase (AChE) inhibitory activities. The combination of HPLC (DAD) and Q-TOF technique could give excellent separating and good structural characterization abilities which make it suitable to analyze complex multi-herbal extracts in TCM. The aim of this study was to develop a HPLC (DAD)/ESI-Q-TOF-MS/MS method for the identification and profiling of pharmacologically active coumarin glycosides in Mori ramulus refined extracts for used in TCM. This HPLC (DAD)/ESI-Q-TOF-MS/MS method provided a rapid and accurate method for identification of coumarin glycosides—including new natural products described here for the first time—in the crude extract of *M. alba* L. In the course of this project, two novel natural products moriramulosid A (umbelliferone-6-β-d-apiofuranosyl-(1→6)-β-d-glucopyranoside) and moriramulosid B (6-[[6-*O*-(6-deoxy-α-l-mannopyranosyl)-β-d-glucopyranosyl]oxy]-2*H*-1-benzopyran-1-one) were newly discovered and the known natural product Scopolin was identified in *M. alba* L. for the first time.

## 1. Introduction

A pathological high xanthine oxidase (XO, EC 1.1.3.22) activity is strongly correlated to hyperuricemy and gout [1]. The prevalence of this disease is 2% to 9 % depending on age and gender and increases continuously in industrialized countries [2]. The pathological symptoms of gout emerge from the extracellular precipitation of monosodium urate crystals in different tissues (e.g., joints) followed by an inflammatory response [2,3]. An anti-hyperuremic therapy often includes the application of XO inhibitors like allopurinol. Upon reaction with the enzyme, allopurinol is oxidized to oxypurinol [2]. Whereas allopurinol is a weak competitive XO inhibitor, oxypurinol exhibits a strong non-competitive inhibitory effect [3]. Unfortunately, the use of the purine analog allopurinol in gout therapy shows adverse effects by inhibiting other enzymes involved in purine metabolism, making the search for alternative XO inhibitors necessary [2].

In this context, several ethnopharmacological approaches have been described [1,4], finding gallic and ellagic acids as well as several flavonoids as inhibitors of XO. Recently, testing of the pharmacological potential of Mediterranean plants by the consortium ‘Local Food-Nutraceuticals’ also included XO inhibitory studies [5]. For example, in Mediterranean traditional medicine olive leaf (*Olea europaea* L.) preparations such as aqueous decocts are used against gout and hypertension [6].

In Traditional Chinese Medicine, Mori ramulus (Chin.Ph.)—the dried twigs of *Morus alba* L.—are extensively used as an antirheumatic [7] agent. Just as several medical plants traditionally used for gout treatment (e.g., *Erythrina stricta* Roxb., *Cunonia macrophylla* Brongn. & Gris., *Olea europaea* L.) also exhibit antiinflammatory effects [6,8,9,10,11,12], the Mori ramulus drug also finds additional use in asthma therapy [13]. This fact as well as the structural complexity, specialized tissue distribution, and manifold regulatory mechanisms of XO strongly suggest a (patho-)physiological XO function beyond the purine metabolism [6].

Many potentially active constituents of *M. alba* such as flavonoids [14], benzofuran derivatives [15], stilbenes [16] and coumarins [17] have been identified in this herbal drug. Coumarins, one of these groups of bioactive constituents, have been demonstrated to possess anti-inflammatory [18], antiplatelet aggregation [19], antitumor [20], as well as both acetylcholinesterase (AChE) [21] and tyrosinase inhibitory activities [22]. Several methods have been reported for the analysis of natural products such as coumarin glycosides using LC-MS—including ion trap—and Q-TOF mass spectrometry [23]. The combination of HPLC (DAD) and Q-TOF technique could give excellent separating and good structural characterization abilities which make it suitable to analyze complex extracts in TCM [24,25,26]. The aim of this study was to develop a HPLC(DAD)/ESI-Q-TOF-MS/MS method for the identification and profiling of pharmacologically active coumarin glycosides in Mori ramulus refined extracts for used in TCM.

## 2. Results and Discussion

### 2.1. Structural Characterization and Fragmentation Behavior of Compounds **A** and **B** and **C**

**Figure 1:**
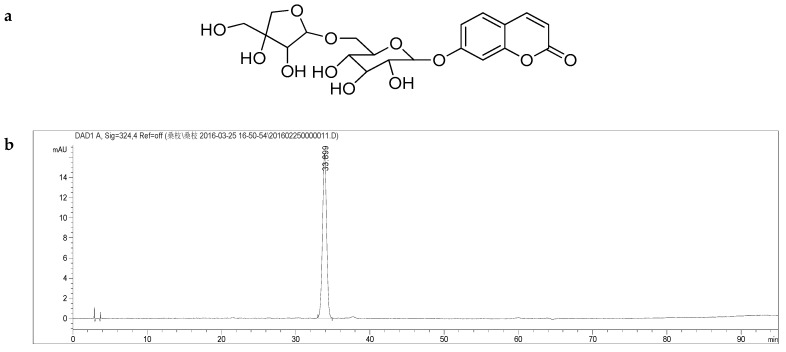
(**a**) Structure of compound **A** C_20_H_24_O_12_, moriramulosid A (umbelliferone-6-β-d-apiofuranosyl-(1→6)-β-d-glucopyranoside); (**b**) HPLC chromatogram of moriramulosid A (umbelliferone-6-β-d-apiofuranosyl-(1→6)-β-d-glucopyranoside). Details of the HPLC-MS method, see main text.

The full-scan mass spectrum of the newly discovered natural product umbelliferone-6-β-d-apiofuranosyl-(1→6)-β-d-glucopyranoside (**A**) contains a [M − H]^−^ ion at *m*/*z* 455.1176, [M + Cl − H]^−^ ion at *m*/*z* 491.0944 and [2M − H]^−^ ion at *m*/*z* 911.2448 in the negative ESI source. The molecular formula of **A** was determined to be C_20_H_24_O_12_ by HRESI-MS analysis [*m*/*z* 455.1176 (M − H)^−^]. In addition, a small abundant ion at *m*/*z* 293.0842 was observed; this suggests Glc-Api residue was present in the structure. In MS/MS spectrum of this ion [M − H]^−^, a product ion at *m*/*z* 161.0235 was observed as a major product ion, resulting from the direct loss of Glc residue from [M − Glc − Api]^−^. The ion at *m*/*z* 161.0235 was very stable and did not yield any further fragmentation. We believe that the Glc residue elimination originate from C-7 of this ion. Consequently, the structure of the novel natural product **A** was identified as shown in Figure 1 (and Figure S1) and was named moriramulosid A.

**Figure 2:**
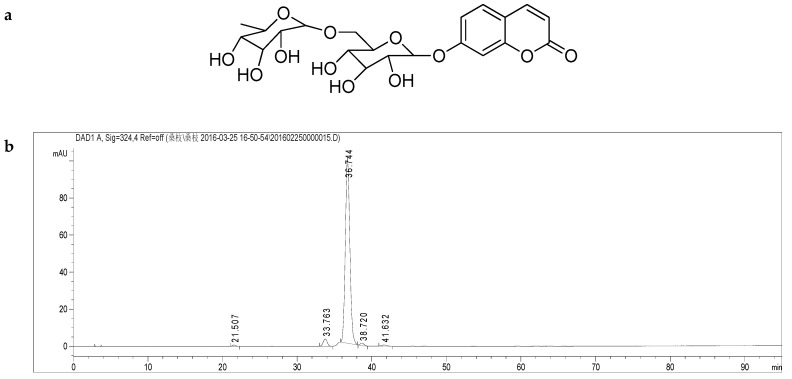
(**a**) Structure of compound **B** C_21_H_26_O_12_, moriramulosid B (6-[[6-*O*-(6-deoxy-α-l-mannopyranosyl)-β-d-glucopyranosyl]oxy]-2*H*-1-benzopyran-1-one); (**b**) HPLC chromatogram of moriramulosid B (6-[[6-*O*-(6-deoxy-α-l-mannopyranosyl)-β-d-glucopyranosyl]oxy]-2*H*-1-benzopyran-1-one). Details of the HPLC-MS method, see main text.

A similar diagnostic fragmentation pattern was observed in the MS and MS/MS spectra of 6-[[6-*O*-(6-deoxy-α-l-mannopyranosyl)-β-d-glucopyranosyl]oxy]-2*H*-1-benzopyran-1-one (**B**). The UV spectra were obtained for the two compounds. The results showed a local absorption maximum around 320 nm for both compounds in the UV spectrum data. Hence, it can be concluded that the fragmentation behavior and UV absorption of these analogue 6-substituent coumarin glycosides are indeed very similar. Consequently, the structure of the novel natural product **B** (Figure 2 and Figure S2) was identified as a second new natural product that was named moriramulosid B.

**Figure 3:**
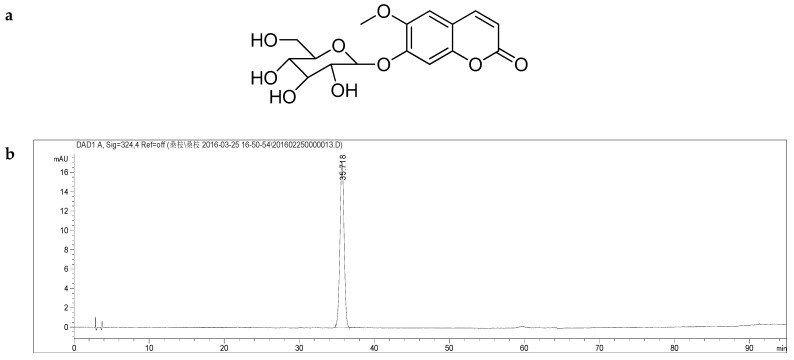
(**a**) Structural formula of compound **C** C_16_H_18_O_9_, Scopolin; (**b**) HPLC chromatogram of Scopolin. Details of the HPLC-MS method, see main text.

In the MS/MS spectrum of **A**, a product ion at *m*/*z* 191 was observed, meanwhile, it is notably that fragment ion at *m*/*z* 176 was labeled as [Y0 − 2H]−. The similar diagnostic fragmentation pattern was observed in the MS and MS/MS spectra of **C**; identified as Scopolin (Figure 3 and Figure S3). Its UV spectrum shows two local absorption maxima: one at ca. 285 nm (band II) and another at ca. 340 nm (band I).

#### 2.1.1. Compound **A**

The ^13^C-NMR and DEPT spectra revealed that compound **A** contains a sugar chain. The ^13^C-NMR spectra of compound **A** displayed three sets characteristic for oxygen bearing methylene—δ_C_ 63.7 (C-6′), δ_C_ 73.5 (C-4″), δ_C_ 68.0 (C-5″)—seven methyne sets and one quaternary carbon atom (see Table 1). Combined with the ^1^H-NMR spectral data, it can be deduced that A contains one hexose group and one apiose group. For the aglycone part of A, the presence of two singlets at δ_H_ 6.34 (d, 9.5 Hz) and δ_H_ 8.00 (d, 9.5 Hz) in the ^1^H-NMR spectra and four singlets at δ_C_ 160.7, δ_C_ 155.4, δ_C_ 144.6, δ_C_ 113.8 in the ^13^C-NMR spectra, were in accord with substitution of benzopyranocoumarion. The presence of three aromatic protons at δ_H_ 7.04 (d, 2.2 Hz), δ_H_ 7.04 (dd, 9.3 Hz, 2.3 Hz), δ_H_ 7.66 (d, 9.3 Hz) assignable to the benzopyranocoumarion structural unit is a typical AMX coupled system, and clearly indicates a C-6 monosubstituted coumarin. The ^13^C-NMR signals of C-6 and C-6′, and ^1^H-NMR signals of H-1′ and H-1″ were assigned on the basis of HMBC connectivity observed for the sugar unit connecting with C-6 aglycone, which was shown by H-1′ and C-7, and H-1″ and C-6′, having long-distance correlations.

#### 2.1.2. Compound **B**

The NMR data of compound **B** are similar to those for compound **A**. **B** also contains a benzopyranocoumarion structural moiety: δ_H_ 6.35 (1H, d, 9.5 Hz), δ_H_ 8.01(1H, d, 9.5 Hz), δ_C_ 160.8, and a typical AMX coupled system: δ_H_ 7.66 (1H, d, 8.4 Hz), δ_H_ 7.03(1H, dd, 8.5 Hz, 2.4 Hz), δ_H_ 7.04 (1H, d, 2.4 Hz) (see Table 2). There are two hexose moieties in the sugar part, one of which contains a methyl group. The HMBC cross-peaks of H-1′ with C-7 and H-1″ with C-6′ indicated that the sugar unit is connected with C-6 aglycone.

#### 2.1.3. Compound **C**

The ^13^C-NMR and DEPT spectra revealed that compound **C** contains a monosaccharide, and the glycosyl group has six carbon signals. The ^13^C-NMR spectra of compound **C** displayed one oxygen bearing a methylene characteristic set—δ_C_ 61.1 (6′-C)—and five methyne sets. Combined with the ^1^H-NMR spectral data, it can be concluded that C contains one glucose group. For the aglycone part of C, the presence of two singlets at δ_H_ 6.33 (1H, d, 9.5 Hz) and δ_H_ 7.97 (1H, d, 9.5 Hz) in the ^1^H-NMR spectra and one carbonyl carbon singlet at δ_C_ 160.7 in the ^13^C-NMR spectra, are in accordance with substitution of benzopyranocoumarion. The presence of singlets of a methoxy group at δ_H_ 3.83 and of an aromatic single hydrogen at δ_H_ 7.30, δ_H_ 7.16 in the ^1^H-NMR spectra, demonstrates that C-5 and C-6 of the benzopyranocoumarion are substituted (see Table 3).

Based on these data, compound **C** was identified as Scopolin, a previously known natural product that was identified as a constituent of *Morus alba* L. for the first time in the present study.

### 2.2 Anti-Hyperuricemic Activity In Vivo

In order to study the effect of the Mori ramulus refined extract ZY1402-A on the serum uric acid levels, in vivo experiments in a mouse model were performed. Therefore, 32 adult male SPF Kunming mice (body weight each between 18 and 22 g) were randomly divided into four groups (*n* = 8), namely the healthy control group (C), the placebo model group (M), the allopurinol positive control group (A), and the group treated with the Mori ramulus refined extract ZY1402-A. All mice were treated by intragastric administration (ig) daily (at 9:00 am) for 8 days. All groups with the exception of the healthy control group (C) were treated (ig) with 300 mg/kg potassium oxonate 1 h before the respective treatment was administered. Blood was collected from the posterior venous plexus of the eye after 1 h of administration of the respective treatment on the 8th day. Subsequently, serum was taken after centrifugation for measuring the levels of serum uric acid (S_UA_). For further details see Section 4.5.

**Figure 4:**
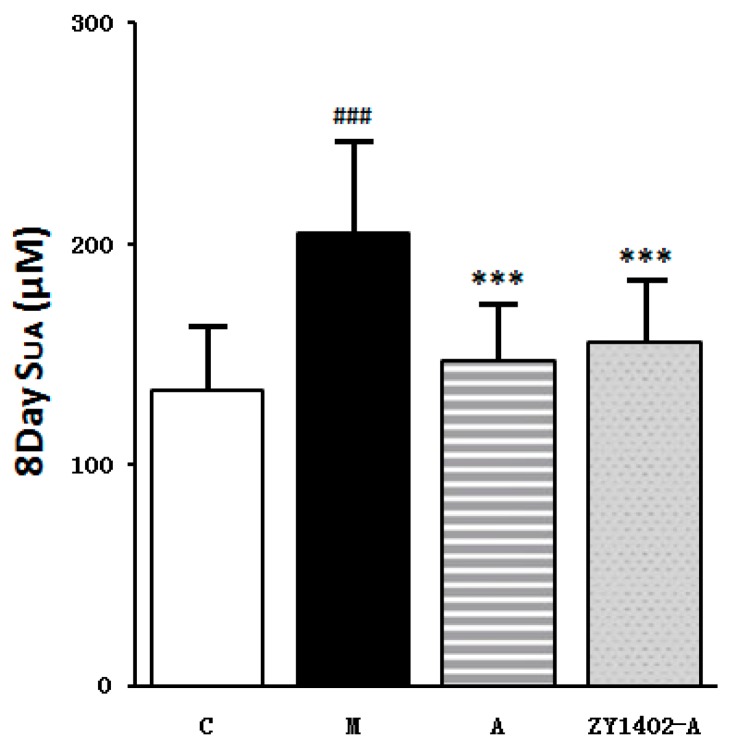
The effect of the Mori ramulus refined extract ZY1402-A on serum uric acid levels in the in vivo mouse model (*n* = 8; ^###^ *p* < 0.001 compared to C; *** *p* < 0.001 compared to M). All data are given as X ± SD. Healthy control group (C), placebo model group (M), allopurinol positive control group (A).

Compared with the healthy control group the serum uric acid levels of the placebo model group were significantly increased. Compared with the placebo model group, both the serum uric acid levels of the positive control group (5 mg/kg allopurinol) and the Mori ramulus refined extract (ZY1402-A) treatment group and were significantly reduced (Figure 4).

## 3. Conclusions

In the present study, two novel natural products from the class of coumarin glycosides—moriramulosid A and B—were isolated and identified for the first time from an ethanol extract of Mori ramulus (Chin.Ph.). Said extract was characterized using negative ion HPLC/ESI-Q-TOF-MS/MS spectra in combination to UV-DAD. This HPLC/ESI-Q-TOF-MS/MS method provided a rapid and accurate method for identification of coumarin glycosides in crude extract from *M. alba* L.

The accompanying mouse model experiments for measuring the anti-hyperuricemic activity of the Mori ramulus refined extract (ZY1402-A) in vivo demonstrate that this extract can reduce the serum uric acid levels of SPF Kunming mice significantly.

## 4. Experimental

### 4.1. Extract Preparation, Reagents, and Chemicals

**Figure 5:**
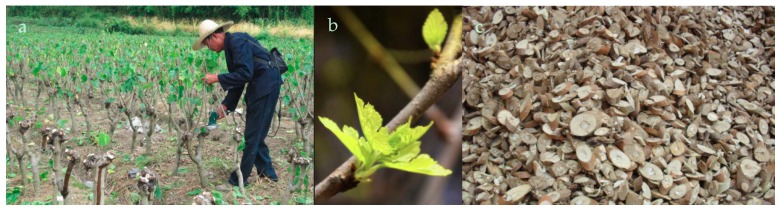
Photos taken at a GAP cultivation site for *Morus alba* L. in Yunnan, China. (**a**) Farmer harvesting *Morus alba* twigs; (**b**) Young leaved sprouting on the *Morus alba* twigs in spring; (**c**) Mori ramulus drug in market form. Although more than 10,000,000 tons of mulberry twigs are produces in the Peoples Republic of China annually, only a small percentage is used as medicine, whereas most is treated as agricultural waste or as firewood.

Mori ramulus (Chin.Ph.)—the dried twigs of *Morus alba* L.—(batch No. 20150113-2) were purchased via Chuxiongtengyang Chinese Herbal Medicine Corporation, from Good Agricultural Practice (GAP) cultivation sites (Figure 5) in Yunnan, China. The plant material was taxonomically identified by the author Houhong He and a voucher specimen (accession No. 20150113) was deposited at the herbarium of Zhejiang CONBA Pharmaceutical, China.

1002.8 g of dried and powdered Mori ramulus (Chin.Ph.) drug were soaked with 60% ethanol (Carl Roth, Karlsruhe, Germany) overnight, and subsequently extracted twice with 7.0 L of 60% ethanol under reflux for 2 h each. After filtration, the liquid extract was evaporated to dryness under reduced pressure resulting in 5.127 g of dry extract residue, which was subsequently dissolved in 90% ethanol. This ethanol solution was precipitated by adding water in order to obtain the aqueous solution of its water soluble constituents. These were subsequently adsorbed to HPD-100 macroporous resin (Cangzhou Bao’en Adsorbing Material Technology, Cangzhou, China), which was eluted with 25% ethanol. Finally, this eluent was evaporated to dryness under reduced pressure, thus yielding the final Mori ramulus (Chin.Ph.) refined extract that was named ZY1402-A.

HPLC grade acetonitrile, methanol, and analytical grade CH_3_COOH were utilized for HPLC analysis. Three compounds **A**, **B**, and **C** were isolated and purified from the above described refined extract of Mori ramulus (Chin.Ph.). Their structures were determined by the analysis of UV, NMR, MS spectra and compared with previous literature. The purities of isolates were over 95%, determined by HPLC/DAD analysis based on a peak area normalization method. The standard solution of each compound was prepared by dissolving it in 60% (*v*/*v*) methanol and stored at 4 °C until analysis.

### 4.2. Chromatography

HPLC was performed on an Agilent series 1260 instrument (Agilent, Waldbronn, Germany) equipped with a quaternary pump, a diode-array detector (DAD), an autosampler, and a column compartment. The sample was separated on an Xtimate XB-C18 column (5 µm, 4.6 × 250 mm, Welch Materials, Shanghai, China). The mobile phase consisted of acetonitrile (mobile phase A); water (H_2_O) containing 0.2% (*v*/*v*) CH_3_COOH (mobile phase B); and 5% (*v*/*v*) CH_3_OH (mobile phase C). The flow rate was 1 mL/min, and column temperature was set at 25 °C. The development of the gradient over time is summarized in Table 4. The DAD detector was monitored at 324 nm, and the on line UV spectra were recorded in the range 190–400 nm.

### 4.3. Mass Spectrometry

An Aglient 6530 Q-TOF mass spectrometer (Agilent, Santa Clara, CA, USA) was connected to the Agilent 1260 HPLC instrument via an ESI interface. The acquisition parameters were as follows: drying gas (N_2_) flow rate, 12.0 L/min; temperature, 350 °C; nebulizer, 60 psig; capillary, 4500 V; fragmentor, 175 V; skimmer, 65 V; OCT RF V, 750 V. Each sample was analyzed in both positive and negative ion mode to provide complimentary information for molecular formulae and structural identification. The quasi-molecular ion [M − H]^−^ of interest in the negative ESI mode MS scan was selected as precursor ion and subjected to Target-MS/MS or Auto-MS/MS analyses. The collision energy (CE) was set at 35 V and the mass range recorded *m*/*z* 100–2000.

### 4.4. NMR Spectroscopy

NMR spectra were recorded in deuterated dimethyl sulfoxides (DMSO-d6) or methanol (MeOD) on a Bruker DRX-500 spectrometer (Bruker biospin, Rheinstetten, Germany) operating at 500 MHz for ^1^H and at 125 MHz for ^13^C including Distortionless Enhancement by Polarization Transfer (DEPT-135) measurements. Chemical shifts are persented in ppm downfield of tetramethylsilane. 1D and 2D NMR experiments were recorded using Mest NOVA software (Mestrelab Research, Santiago de Compostela, Spain). 

### 4.5. Anti-Hyperuricemic Activity

In order to study the effect of the Mori ramulus refined extract ZY1402-A on the serum uric acid levels, in vivo experiments in a mouse model were performed based on the mouse model developed by Wang M. et al. 2016 [27]. Therefore, 32 adult male SPF Kunming mice (body weight each between 18 and 22 g) obtained from the Shanghai Jiesijie Experiment Animal (Shanghai, China) were acclimatized for two days under 12 h/day light cycle (environment temperature 25 ± 2 °C) and food and drinking water ad libitum. Mice were randomly divided into four groups (*n* = 8), namely the healthy control group (C), the placebo model group (M), the allopurinol positive control group (A), and the group treated with the Mori ramulus refined extract ZY1402-A. All mice were treated by intragastric administration (ig) daily (at 9:00 am) for 8 days. All groups with the exception of the healthy control group (C) were treated (ig) with 300 mg/kg potassium oxonate 1 h before the respective treatment was administered. Blood was collected from the posterior venous plexus of the eye in mice after 1 h of administration in the eighth day, and serum was taken after centrifugation. The levels of serum uric acid (S_UA_) were measured. All data were expressed by X ± SD. SPSS19.0 one-way analysis of variance (ANOVA) was used to look at the statistical difference between the groups.

All animal maintenance and experimental studies were based on the guidelines of the National Institutes of Health for the Care and Use of Animals of the People’s Republic of China, and were approved by the Experiment Animal Center of Nanjing University.

## Figures and Tables

**Table 1:** ^1^H- and ^13^C-NMR Data for Compound **A**.

No.	^13^C	^1^H
**Benzopyranocoumarion Structural Moiety**		
1	160.7	/
2	113.8	6.34 (1H, d, *J* = 9.5 Hz)
3	144.6	8.00 (1H, d, *J* = 9.5 Hz)
4	130.0	7.66 (1H, d, *J* = 9.3 Hz)
5	113.8	7.04 (1H, d, *J* = 2.2 Hz)
6	160.6	/
7	109.8	7.04 (1H, dd, *J* = 9.3 Hz, 2.3 Hz)
8	155.4	/
9	113.8	/
**Hexose Moiety**		
1′	100.4	5.02 (1H, d, *J* = 7.4 Hz)
2′	76.8	3.45 (1H, d, *J* = 7.0 Hz)
3′	73.8	3.46 (1H, d, *J* = 7.0 Hz)
4′	70.3	3.13 (1H, t, *J* = 9.3 Hz)
5′	76.0	3.26~3.29 (1H, m)
6′	63.7	3.70~3.76 (2H, m)
**Apiose Moiety**		
1″	103.8	4.81 (1H, d, 3.1 Hz)
2″	76.4	3.30~3.33 (1H, m)
3″	79.2	/
4″	73.5	3.59~3.62 (2H, m)
5″	68.0	3.87~3.91 (2H, m)

**Table 2:** ^1^H- and ^13^C-NMR Data for Compound **B**.

No.	^1^H-NMR	^13^C-NMR
**Benzopyranocoumarion Structural Moiety**		
1	/	160.8
2	6.35 (1H, d, *J* = 9.5 Hz)	113.8
3	8.01 (1H, d, *J* = 9.5 Hz)	144.7
4	7.66 (1H, d, *J* = 8.4 Hz)	113.8
5	7.03 (1H, dd, *J* = 8.5 Hz, 2.4 Hz)	103.9
6	/	160.6
7	7.04 (1H, d, *J* = 2.4 Hz)	101.0
8	/	155.4
9	/	113.6
**Hexose moiety**		
1′	5.03 (1H, d, *J* = 7.4 Hz)	100.5
2′	3.51 (1H, d, *J* = 3.4 Hz)	76.9
3′	3.50 (1H, d, *J* = 3.2 Hz)	73.5
4′	3.14~3.16 (1H, m)	70.2
5′	3.26~3.29 (1H, m)	76.0
6′	3.82~3.94 (2H, m)	66.6
1″	4.53 (1H, d, *J* = 1.3 Hz)	101.0
2″	3.45~3.49 (1H, m)	72.3
3″	3.41~3.44 (1H, m)	70.8
4″	3.17~3.20 (1H, m)	68.8
5″	3.30~3.32 (1H, m)	71.1
6″-Me	1.08 (3H, d, *J* = 6.3 Hz)	68.2

**Table 3:** ^1^H- and ^13^C-NMR Data for Compound **C**.

No.	^1^H-NMR	^13^C-NMR
**Benzopyranocoumarion Structural Moiety**		
1	/	160.9
2	6.33 (1H, d, *J* = 9.5 Hz)	110.2
3	7.97 (1H, d, *J* = 9.5 Hz)	144.6
4	7.30 (1H, s)	113.7
5	/	149.4
6	/	150.4
7	7.16 (1H,s)	103.5
8	/	146.5
9	/	112.7
10-OMe	3.83 (3H, s)	56.5
**Hexose Moiety**		
1′	5.09 (1H, d, *J* = 7.4 Hz)	100.2
2′	3.40~3.44 (1H, m)	73.5
3′	3.30~3.33 (1H, m)	77.2
4′	3.17 (1H, t, *J* = 9.0 Hz)	70.1
5′	3.28~3.30 (1H, m)	77.6
6′	3.45~3.48 (1H, m) 3.68~3.71 (1H, m)	61.1

**Table 4:** HPLC gradient.

Time (min)	Mobile Phase A (%)	Mobile Phase B (%)	Mobile Phase C (%)
0~50	4	5	91
50~60	4→6	5	91→89
60~60.01	6→5	5→6	89
60.01~70	5→7	6	89→87
70~80	7→12	6	87→82
80~85	12→17	6	82→77
85~90	17→0	6→100	77→0
90~95	0	100	0

## Supplementary Materials

The following are available online at http://www.mdpi.com/1420-3049/24/3/629/s1. Figures S1–S3.
