# Supplementary material for: Mori Ramulus (Chin.Ph.)—the Dried Twigs of Morus alba L./Part 1: Discovery of Two Novel Coumarin Glycosides from the Anti-Hyperuricemic Ethanol Extract
Source: Molecules. 2019 Feb 11;24(3):629. doi: 10.3390/molecules24030629 (PMC6384676; doi:10.3390/molecules24030629)
Supplement: Supplementary file 1 [file molecules-24-00629-s001.pdf]

molecules

MDPI

Supplement

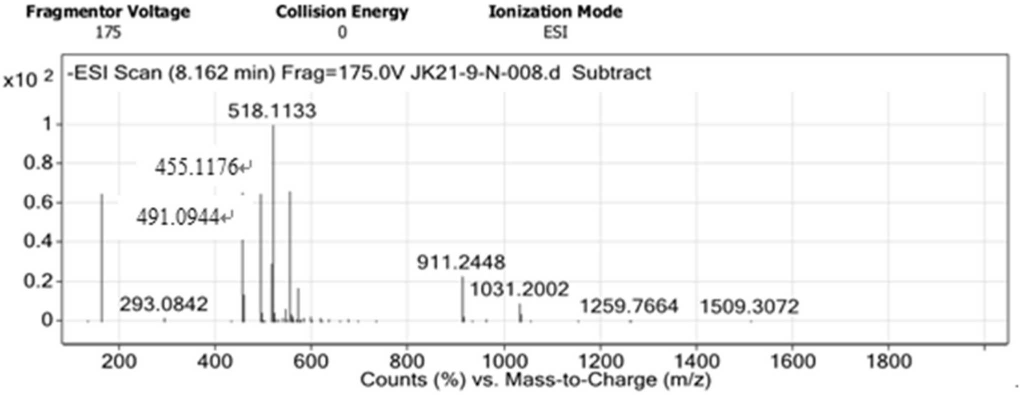

Figure S1c. Primary MS spectrum (negative ion mode) of the moriramulosid A peak in the HPLC chromatogram depicted in (Figure. 1b). Details of the HPLC-MS method see main text.

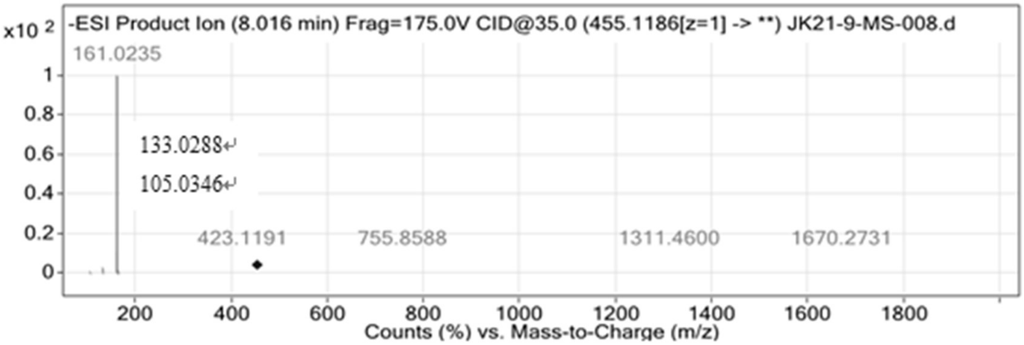

Figure S1d. Secondary MS spectrum (negative ion mode) of the moriramulosid A peak in the HPLC chromatogram depicted in (Fig. 1b). Details of the HPLC-MS method see main text.

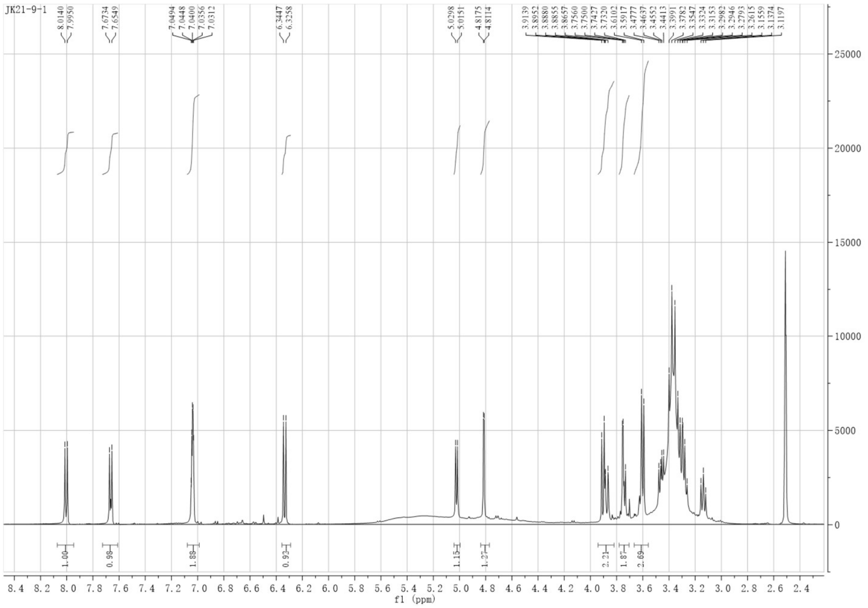

Figure S1e. ^{1}H-NMR of moriramulosid A. Details of the ^{1}H-NMR method see main text.

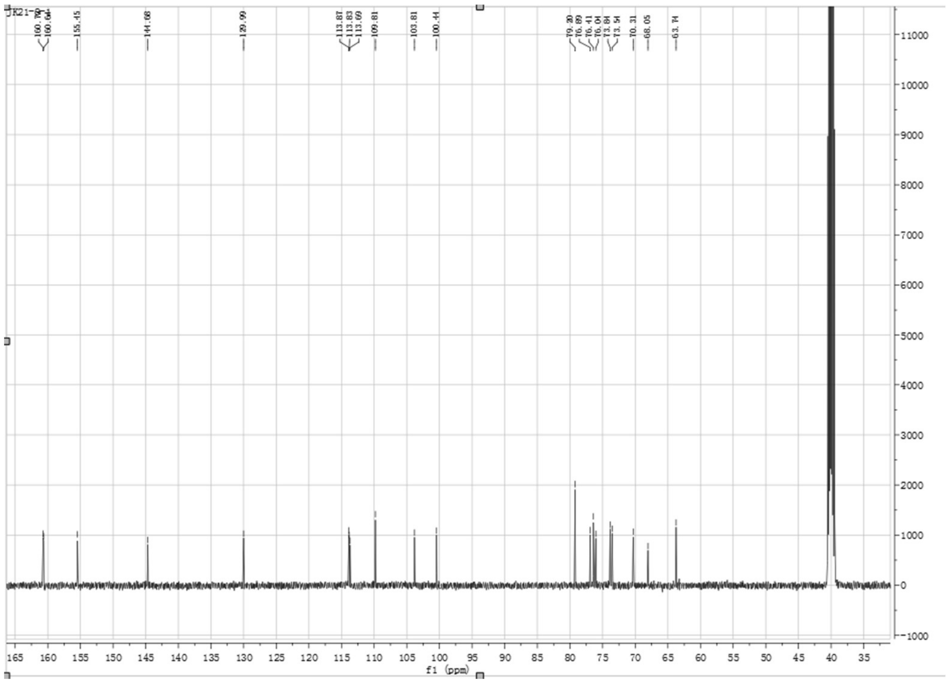

Figure S1f. ^{13}C-NMR of moriramulosid A. Details of the ^{13}C-NMR method see main text.

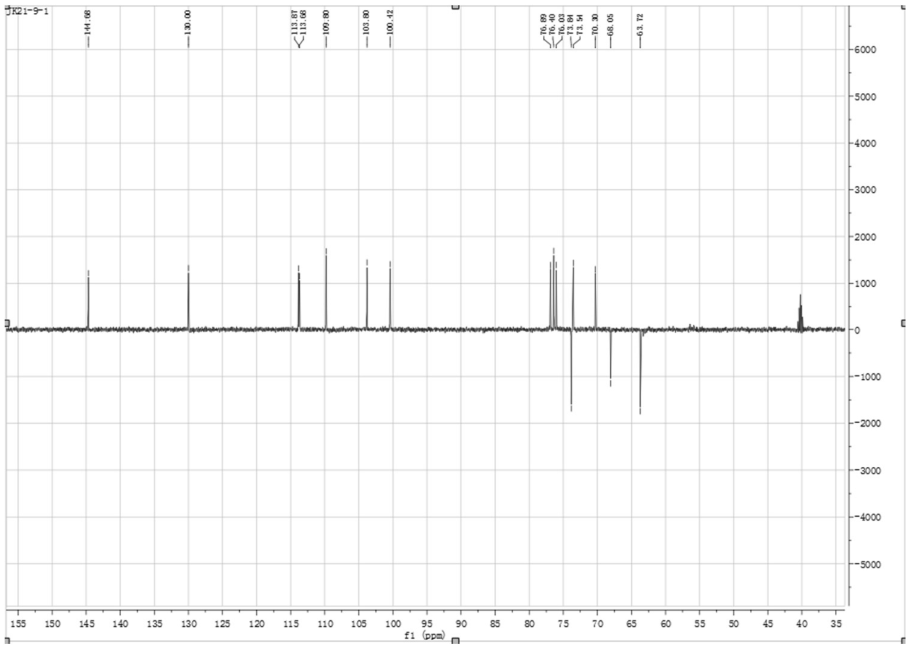

Figure S1g. DPT135 ^{13}C-NMR of moriramulosid A. Details of the DPT135 ^{13}C-NMR method see main text.

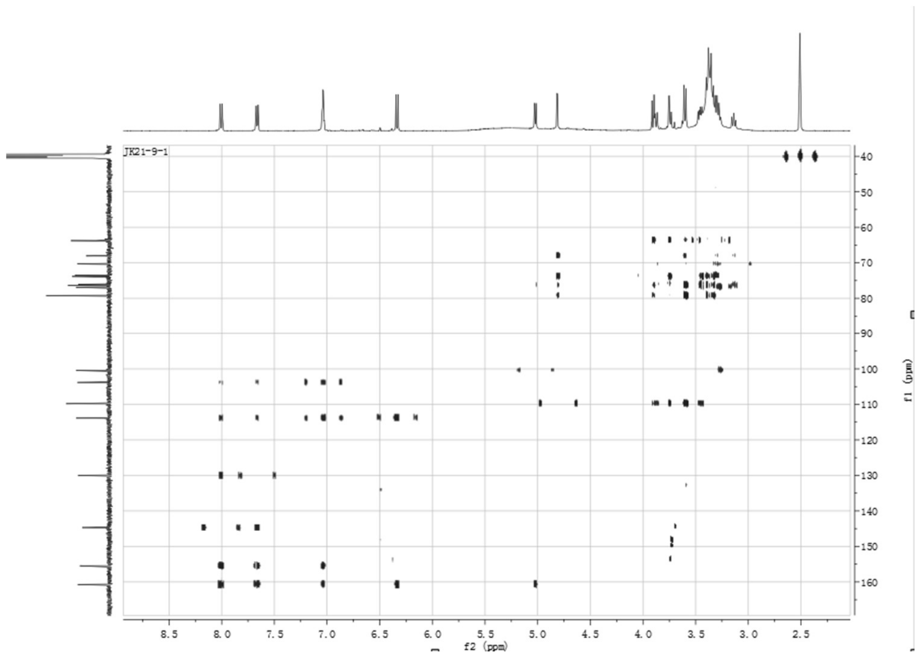

Figure S1h. Heteronuclear multiple-bond correlation spectroscopy (HMBC) NMR of moriramulosid A. Details of the HMBC method see main text.

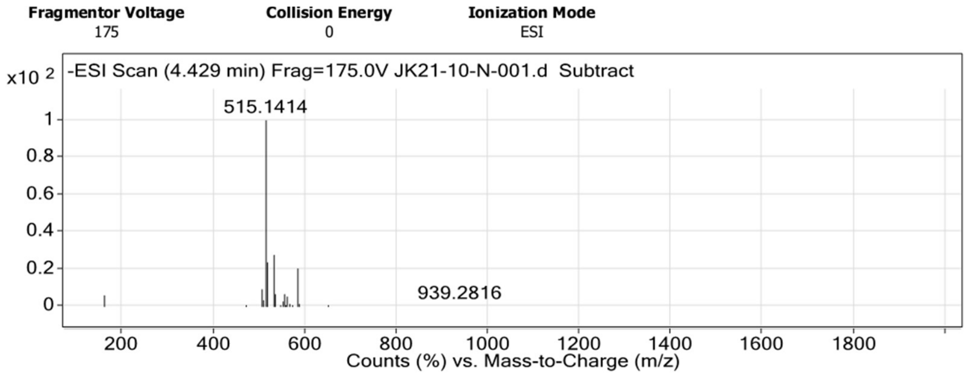

Figure S2c. Primary MS spectrum (negative ion mode) of the moriramulosid B peak in the HPLC chromatogram depicted in (Fig. 2b). Details of the HPLC-MS method see main text.

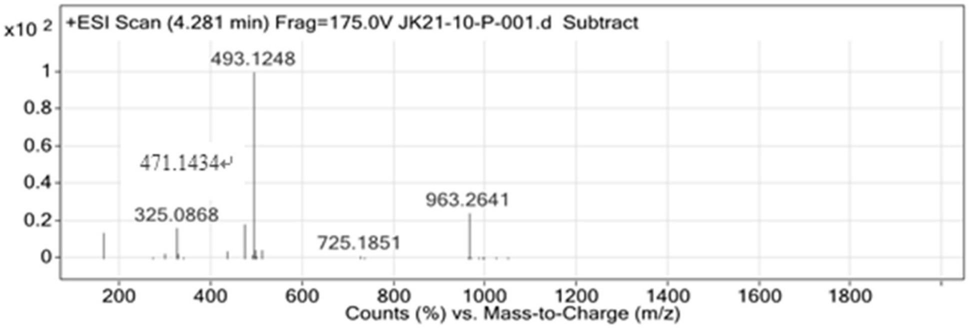

Figure S2d. Primary MS spectrum (positive ion mode) of the moriramulosid B peak in the HPLC chromatogram depicted in (Fig. 2b). Details of the HPLC-MS method see main text.

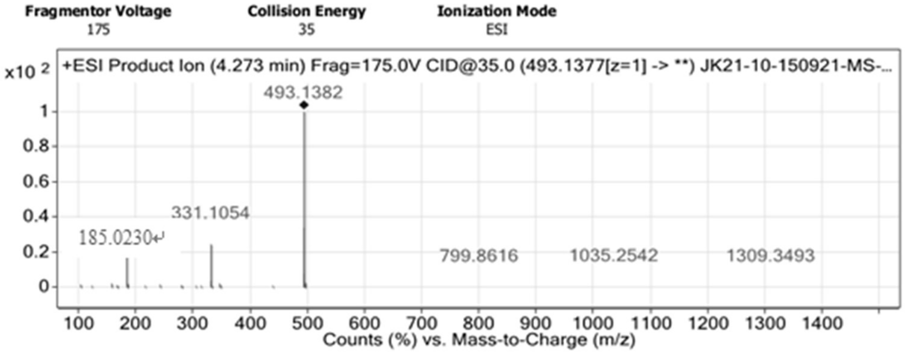

Figure S2e. Secondary MS spectrum (positive ion mode) of the moriramulosid B peak in the HPLC chromatogram depicted in (Fig. 2b). Details of the HPLC-MS method see main text.

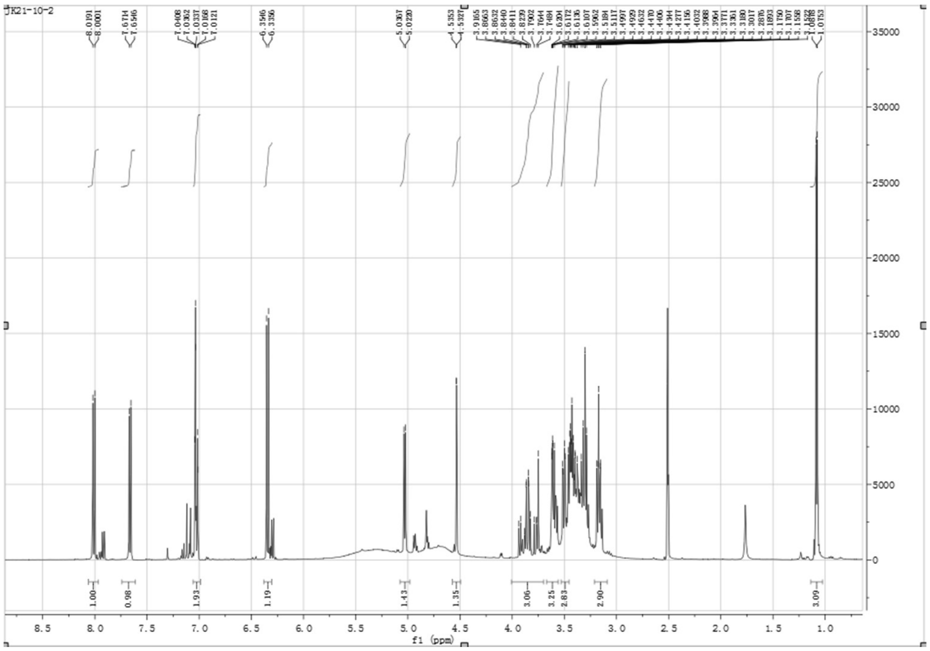

Figure S2f. ^{1}H-NMR of moriramulosid B. Details of the ^{1}H-NMR method see main text.

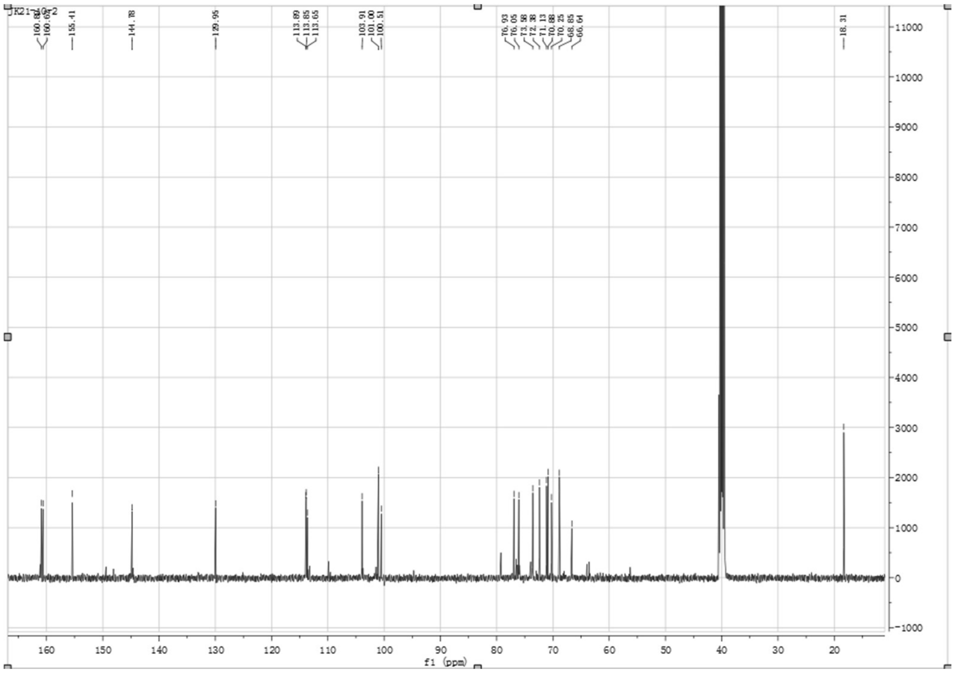

Figure S2g. ^{13}C-NMR of moriramulosid B. Details of the ^{13}C-NMR method see main text.

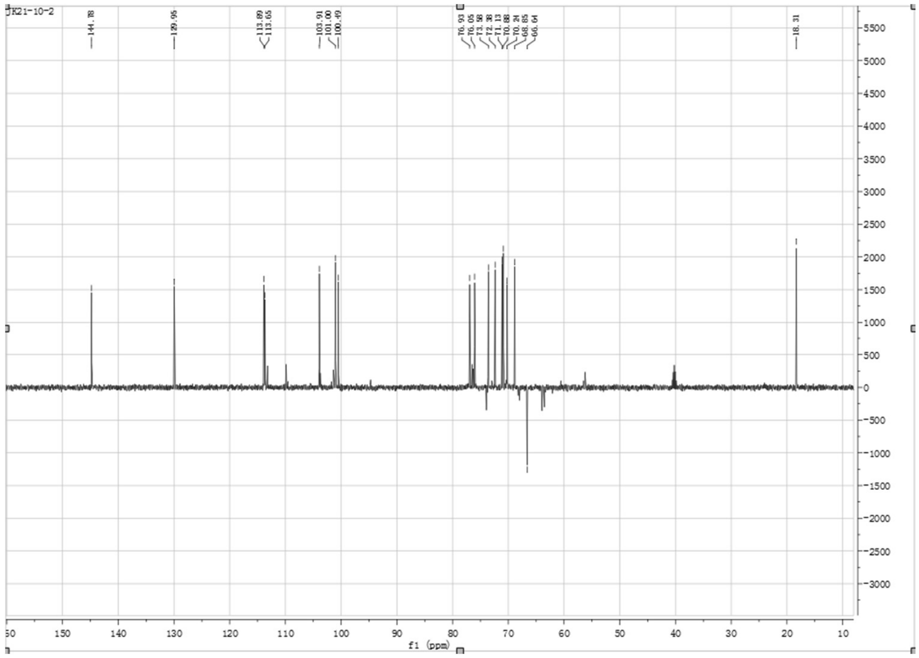

Figure S2h. DPT135 ^{13}C-NMR of moriramulosid B. Details of the DPT135 ^{13}C-NMR method see main text.

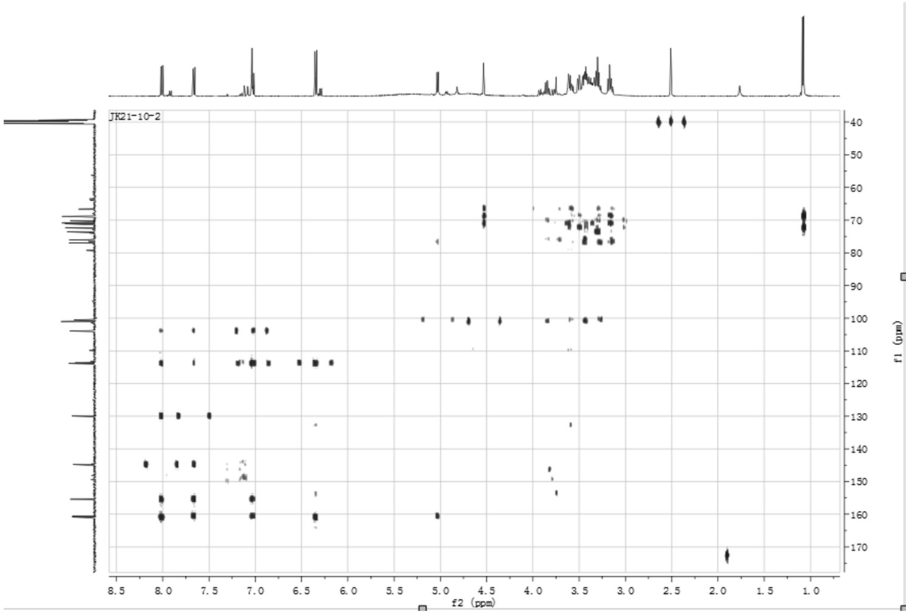

Figure S2i. Heteronuclear multiple-bond correlation spectroscopy (HMBC) NMR of moriramulosid B. Details of the HMBC method see main text.

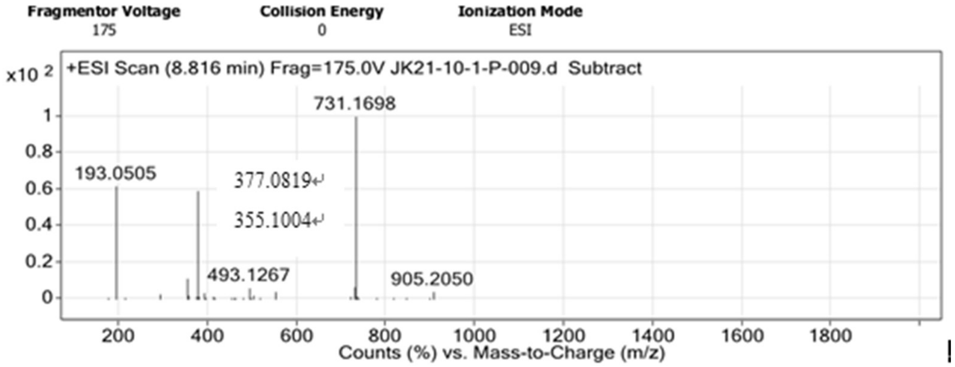

Figure S3c. Primary MS spectrum (positive ion mode) of the Scopolin peak in the HPLC chromatogram depicted in (Fig. 3b). Details of the HPLC-MS method see main text.

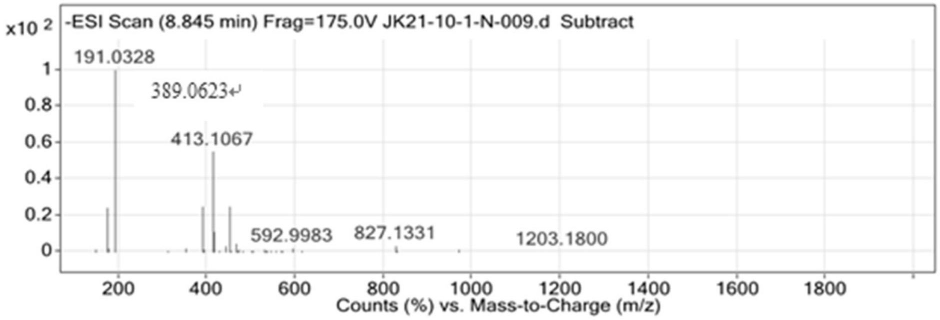

Figure S3d. Primary MS spectrum (negative ion mode) of the Scopolin peak in the HPLC chromatogram depicted in (Fig. 3b). Details of the HPLC-MS method see main text.

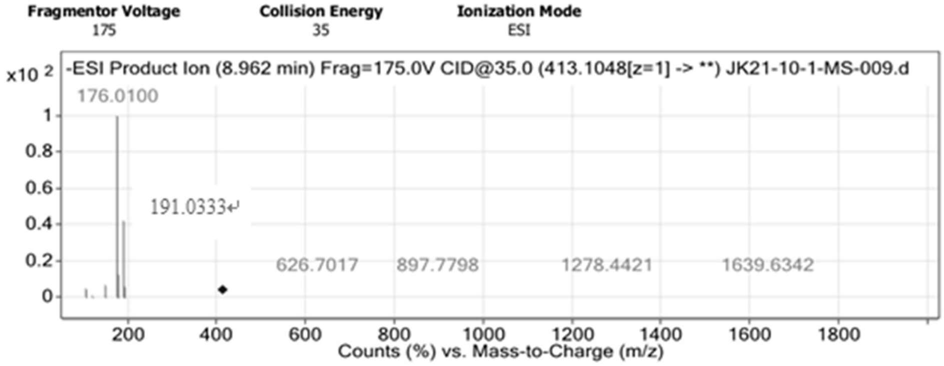

Figure S3e. Secondary MS spectrum (negative ion mode) of the Scopolin peak in the HPLC chromatogram depicted in (Fig. 3b). Details of the HPLC-MS method see main text.

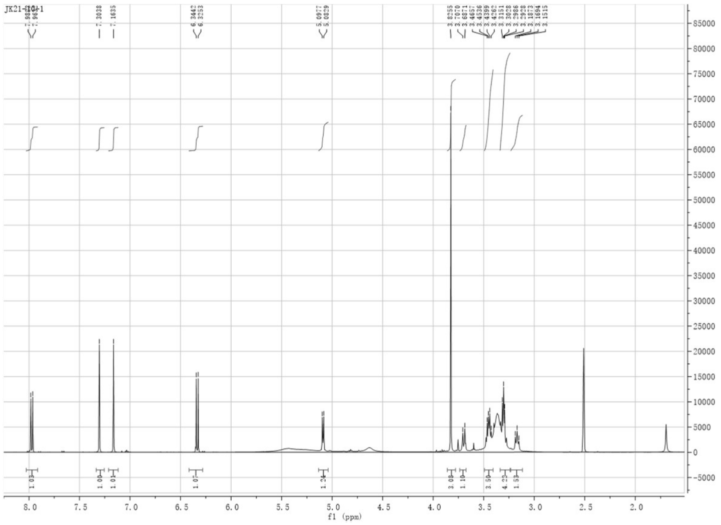

Figure S3f. ^{1}H-NMR of Scopolin. Details of the ^{1}H-NMR method see main text.

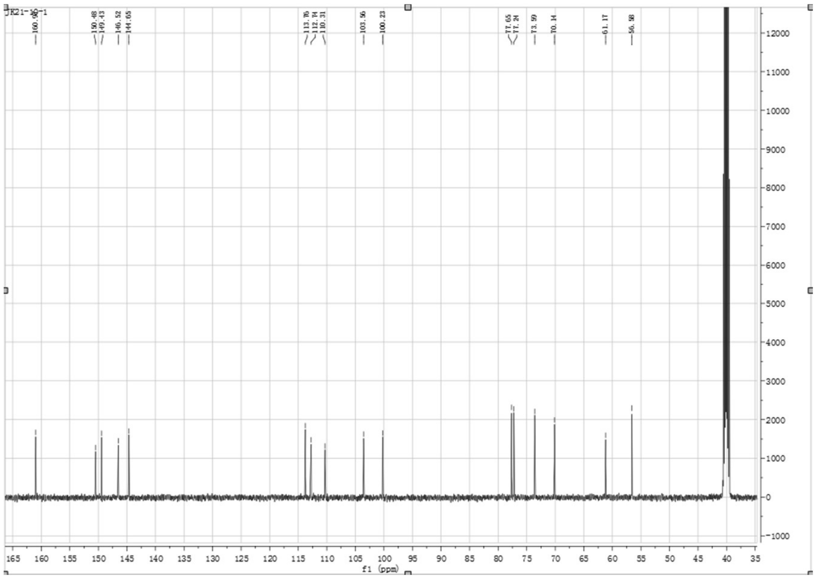

Figure S3g. ^{13}C-NMR of Scopolin. Details of the ^{13}C-NMR method see main text.

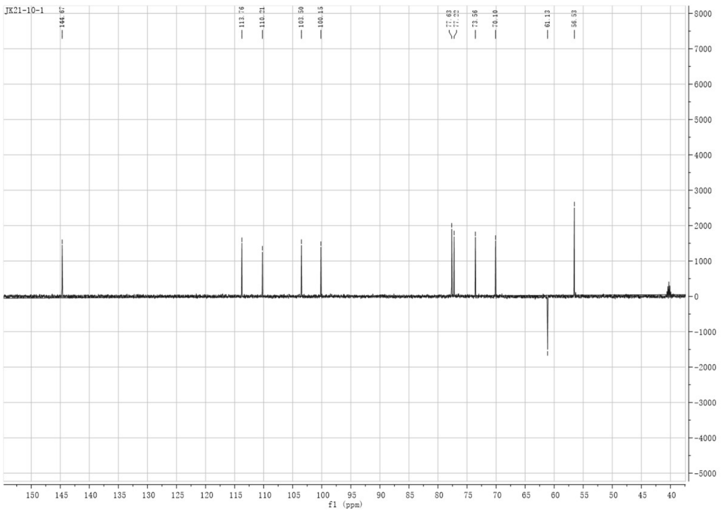

Figure S3h. DPT135 ^{13}C-NMR of Scopolin. Details of the DPT135 ^{13}C-NMR method see main text.
